# Supplementary figures and images for: Regional differences and dynamic evolution of agricultural water resources utilization efficiency in China
Source: PLoS One. 2023 Sep 28;18(9):e0282051. doi: 10.1371/journal.pone.0282051 (PMC10538784; doi:10.1371/journal.pone.0282051)

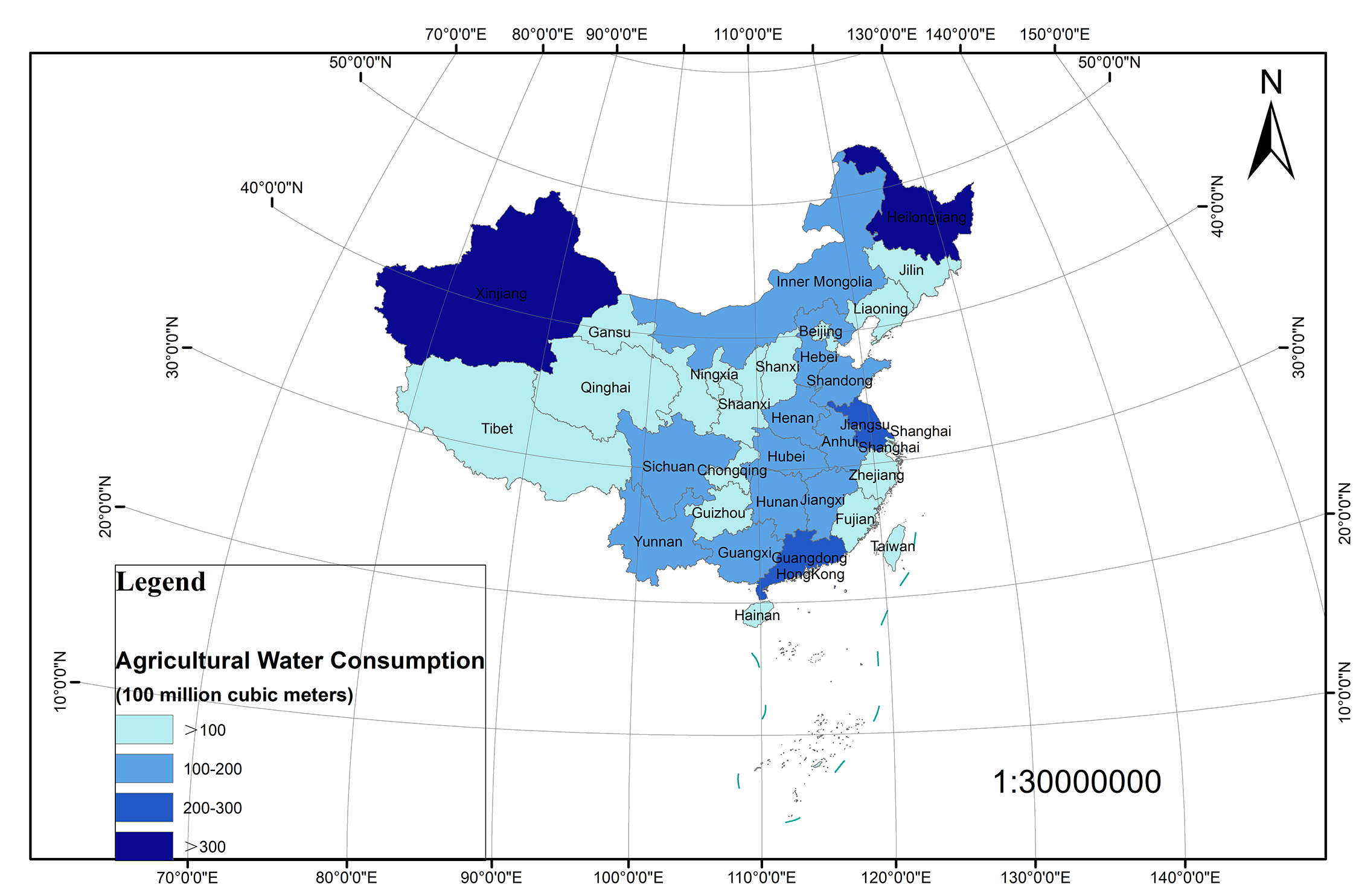

Supplement: S1 Fig — Note:Map created using ArcGIS[10.7]. SHP data downloaded from standard map service system(http://www.resdc.cn/). (PNG) [file pone.0282051.s001.png]

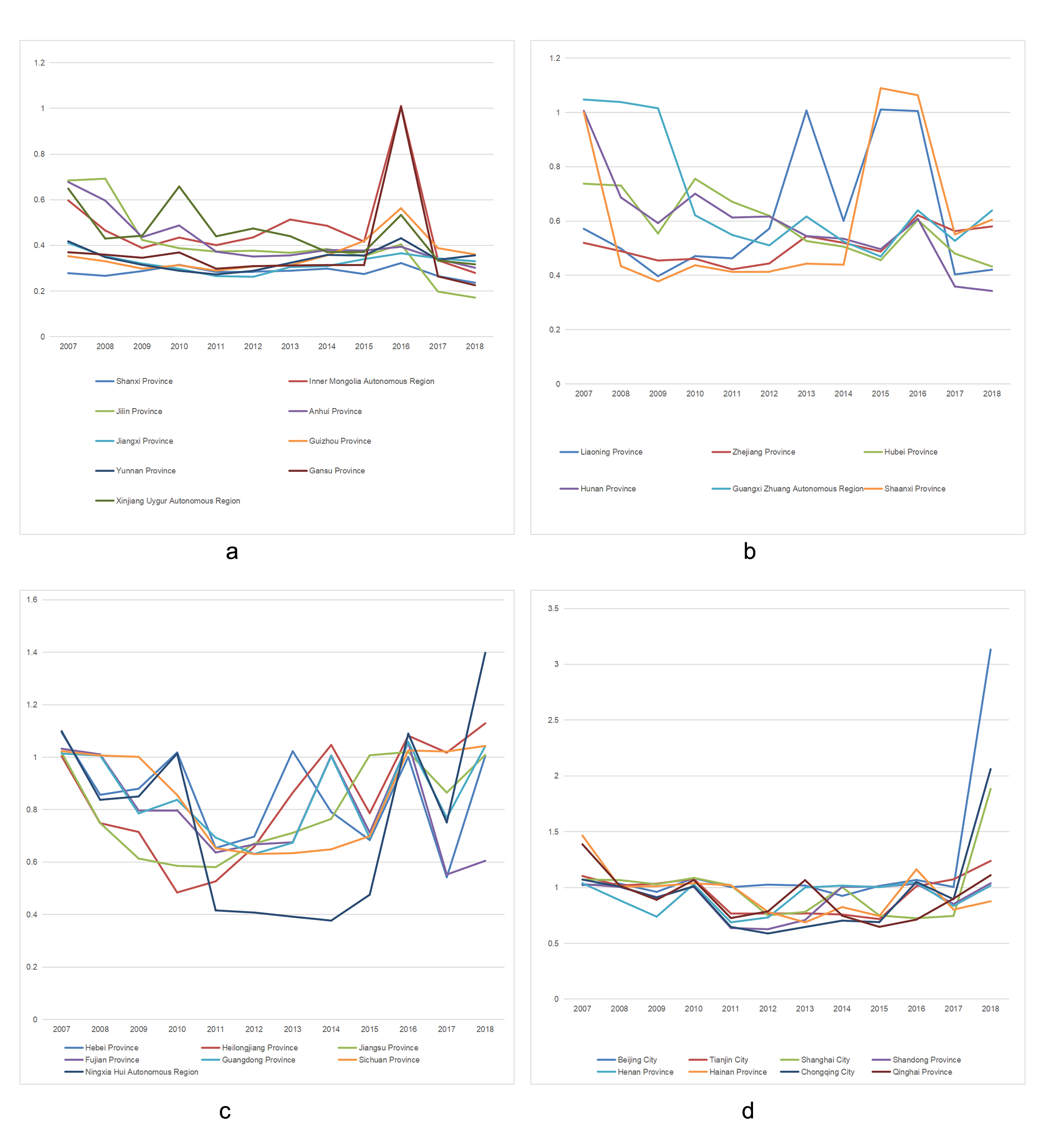

Supplement: S2 Fig — The above four charts are listed seperately according to the average water use efficiency of (a) below 0.5, (b) 0.5–0.7, (c) 0.7–0.9, and (d) 0.9 and above. (PNG) [file pone.0282051.s002.png]

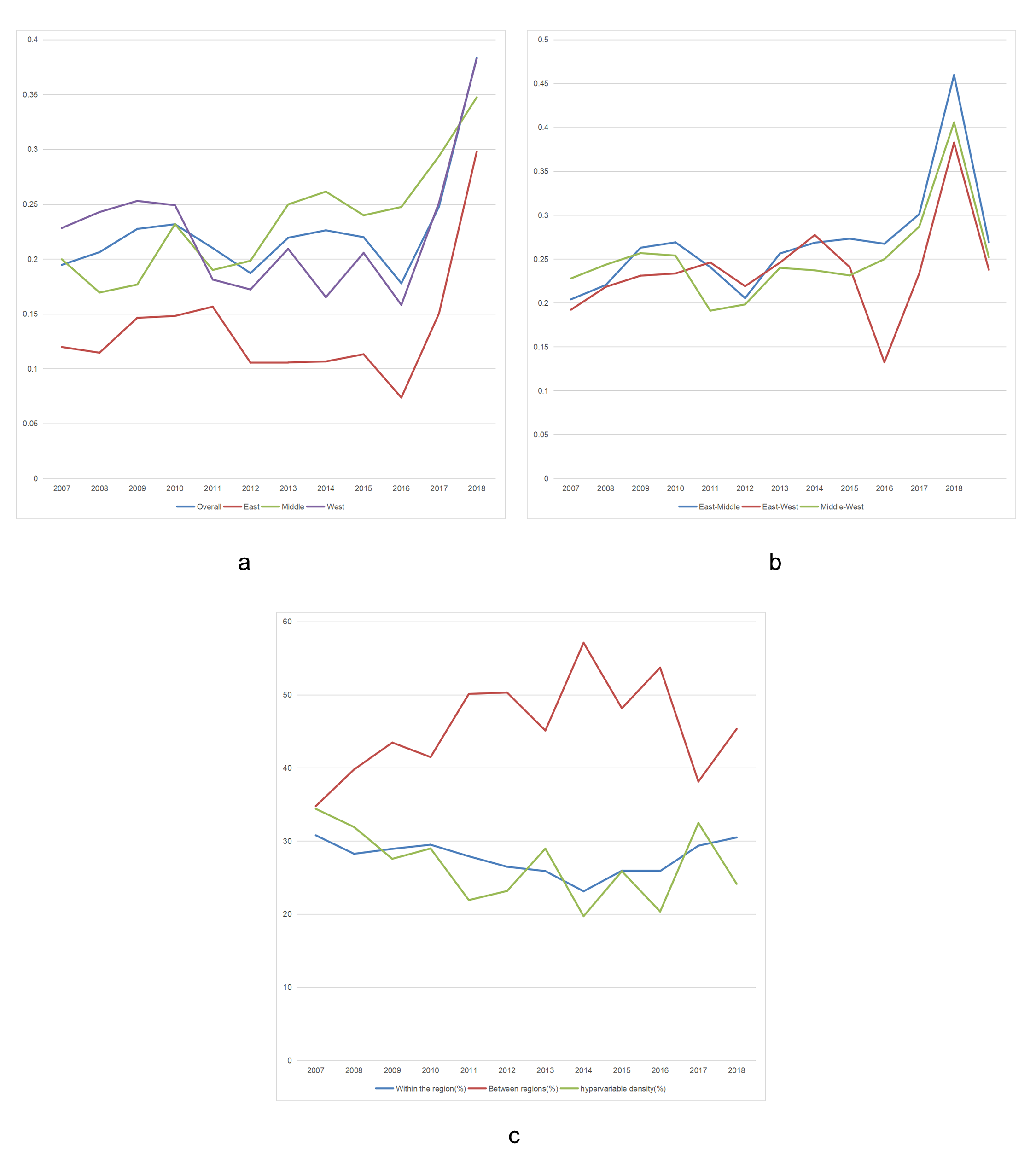

Supplement: S3 Fig — (PNG) [file pone.0282051.s003.png]

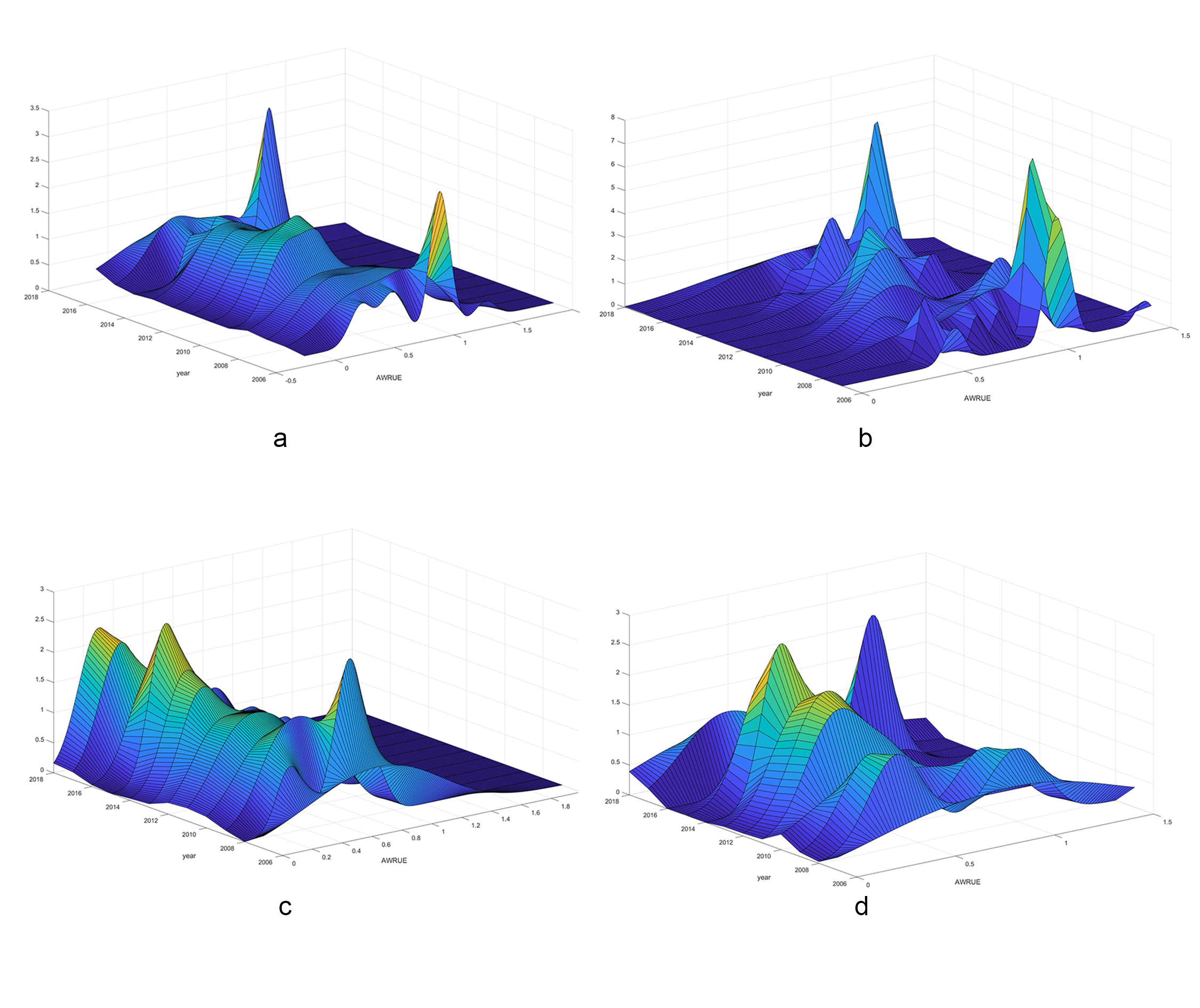

Supplement: S4 Fig — (PNG) [file pone.0282051.s004.png]

## Slide 1
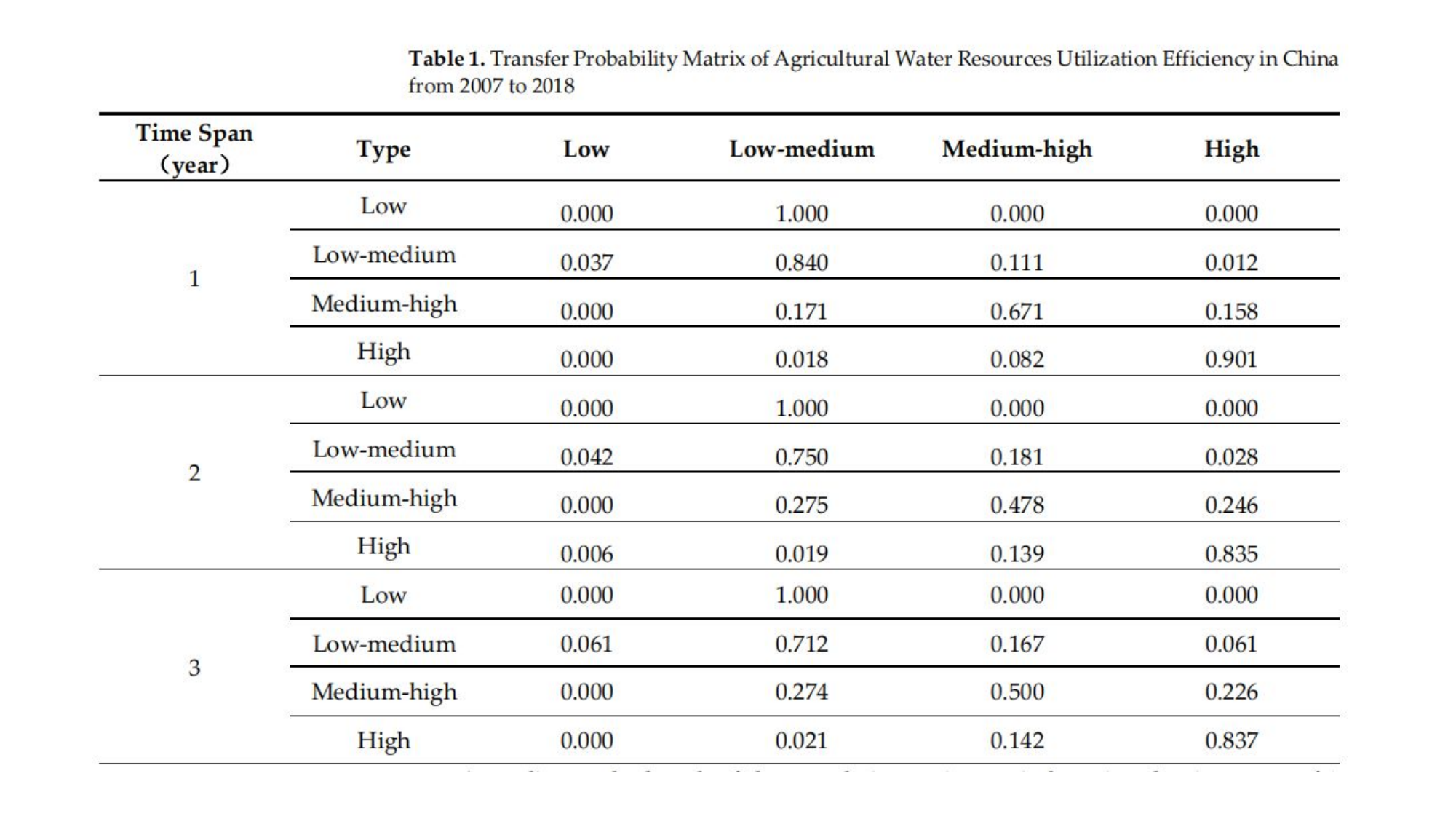

## Slide 2
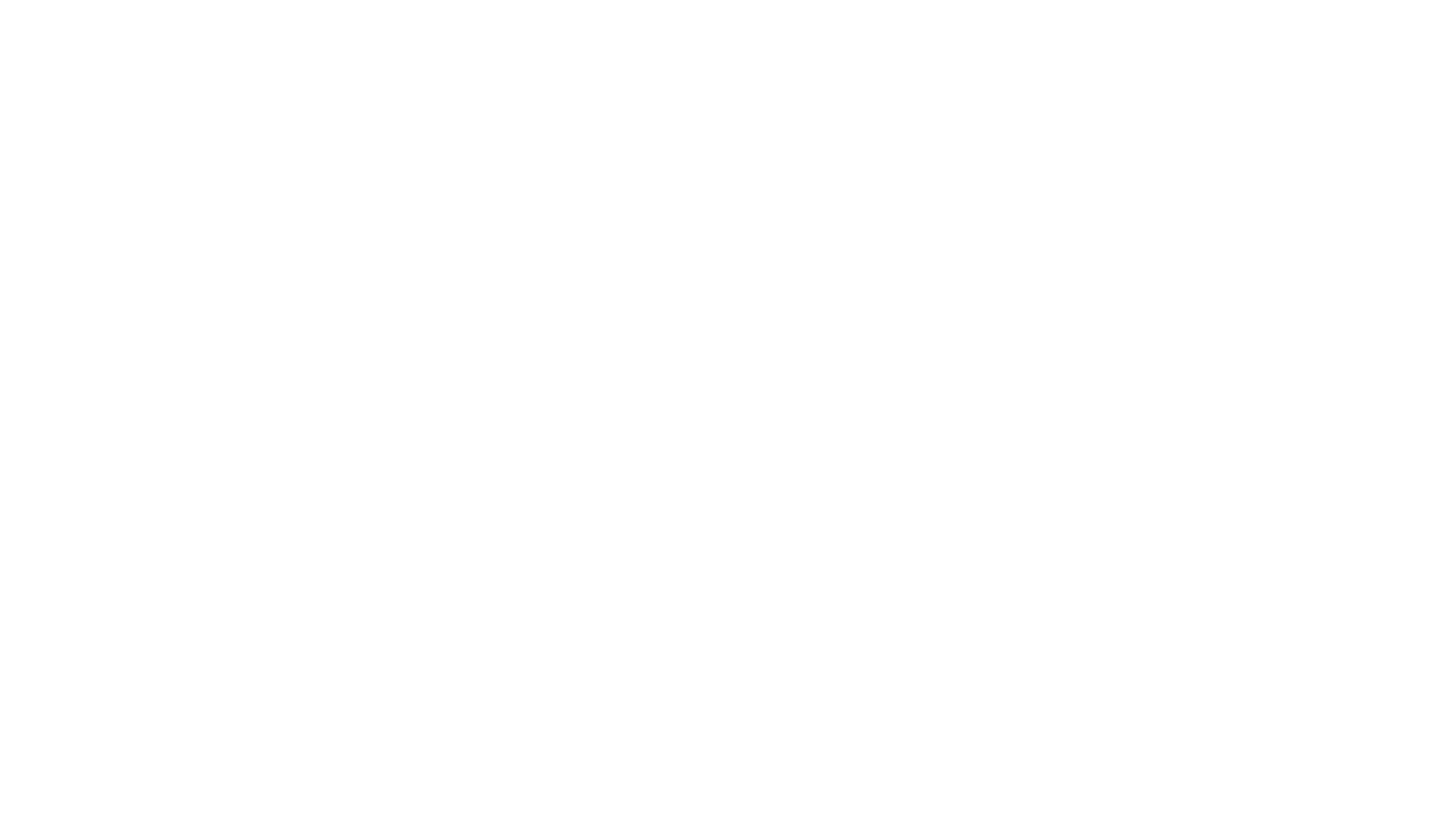

## Slide 3
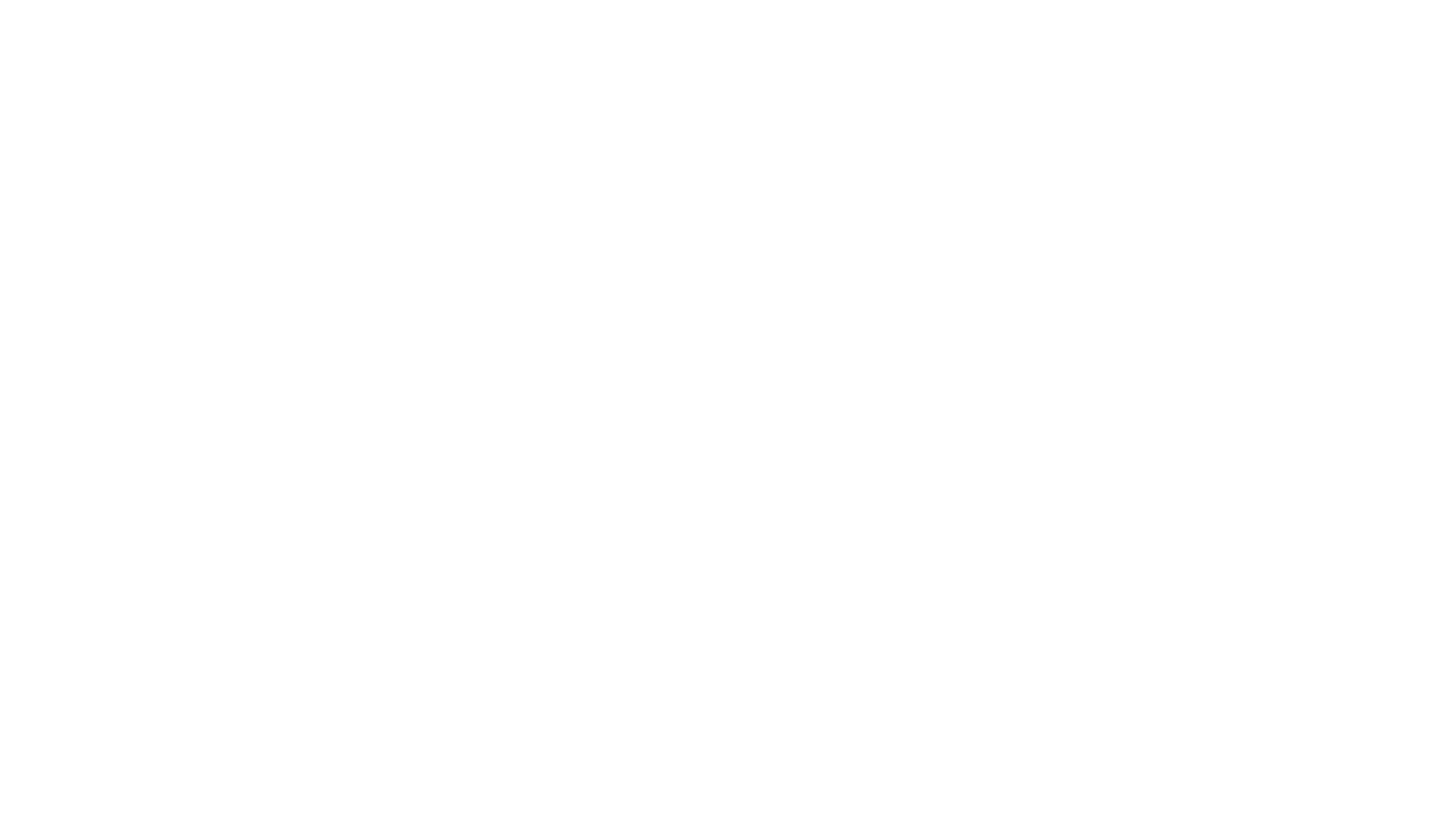

## Slide 4
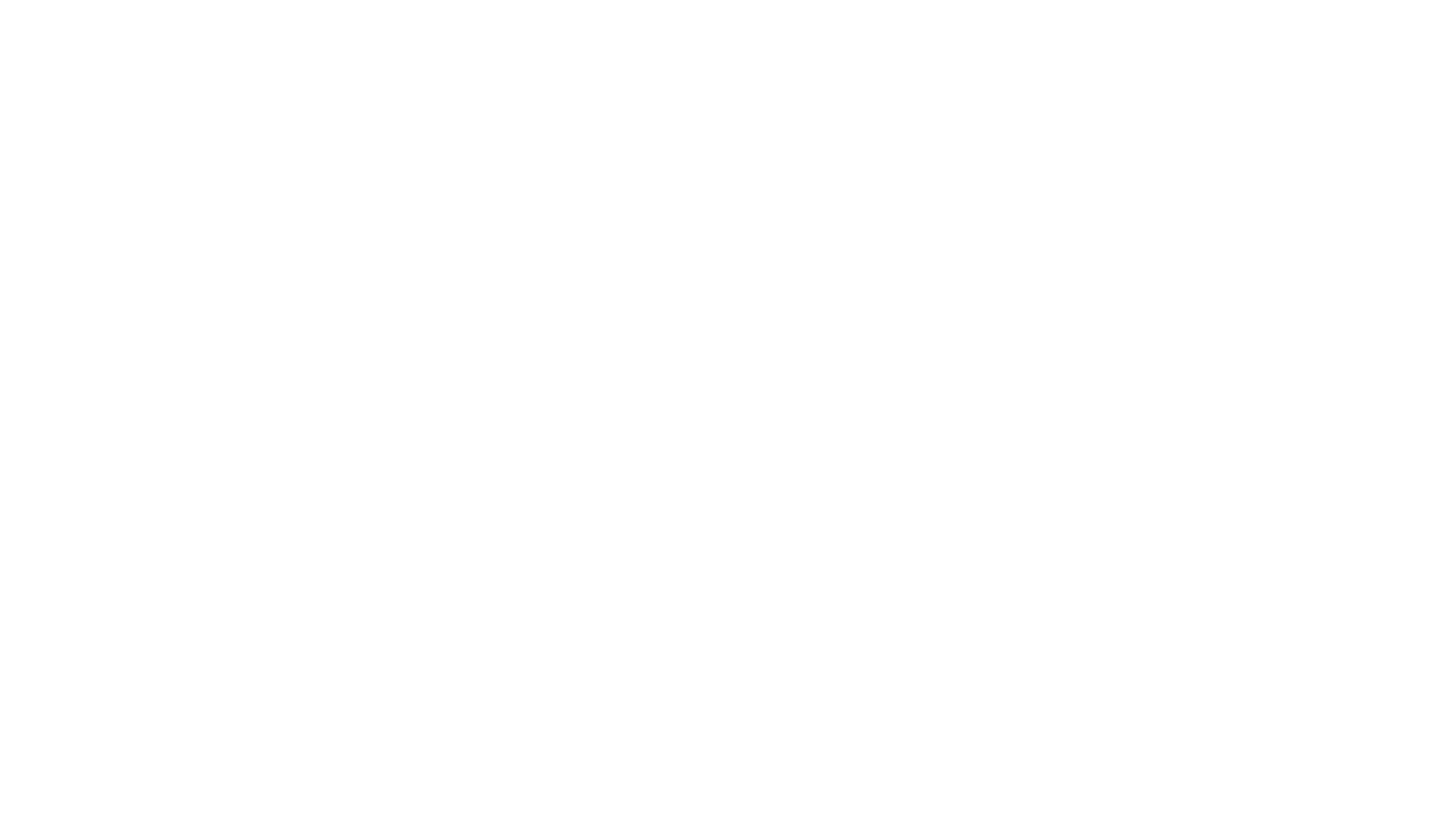

## Slide 5
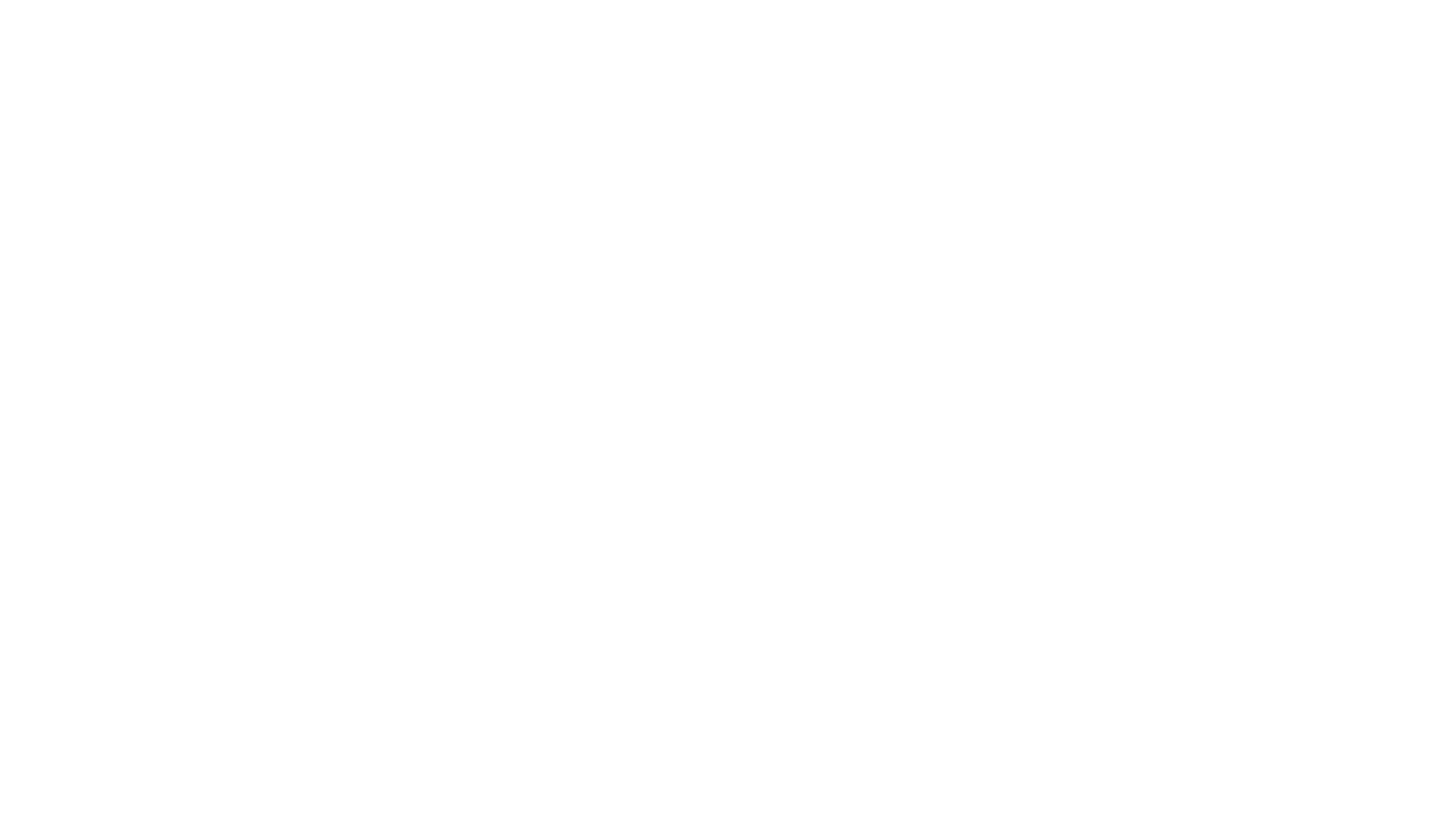

## Slide 6
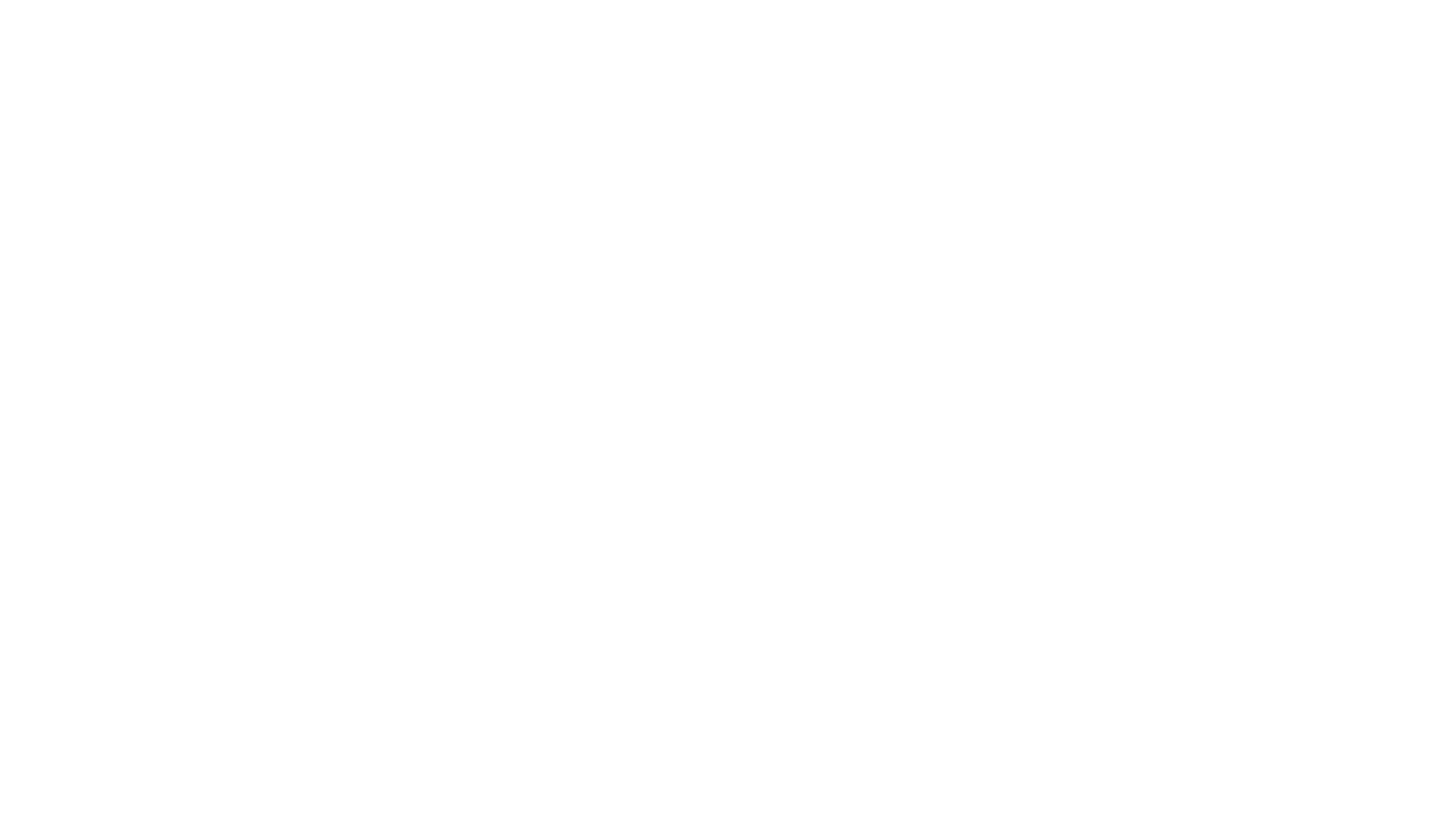

Supplement: S1 Table — (PPTX) [file pone.0282051.s005.pptx]

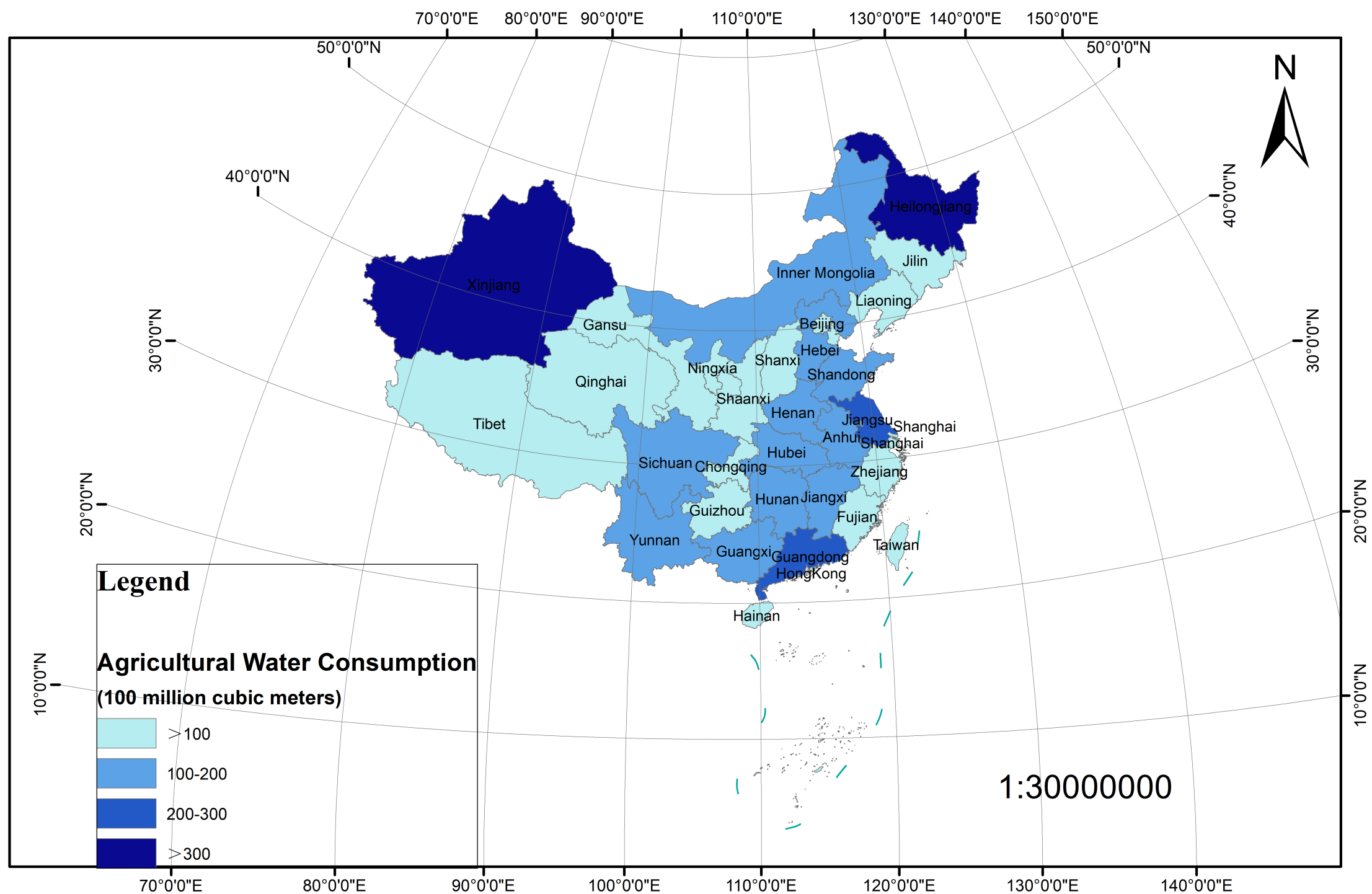

Fig 1.tif

Supplement: S1 Raw images — (PDF) [file pone.0282051.s006.pdf]

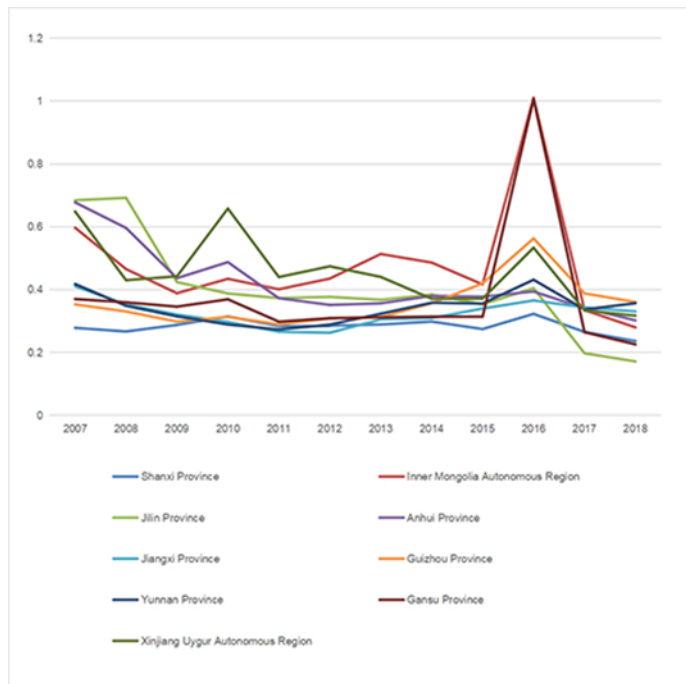

Fig 2a.tif

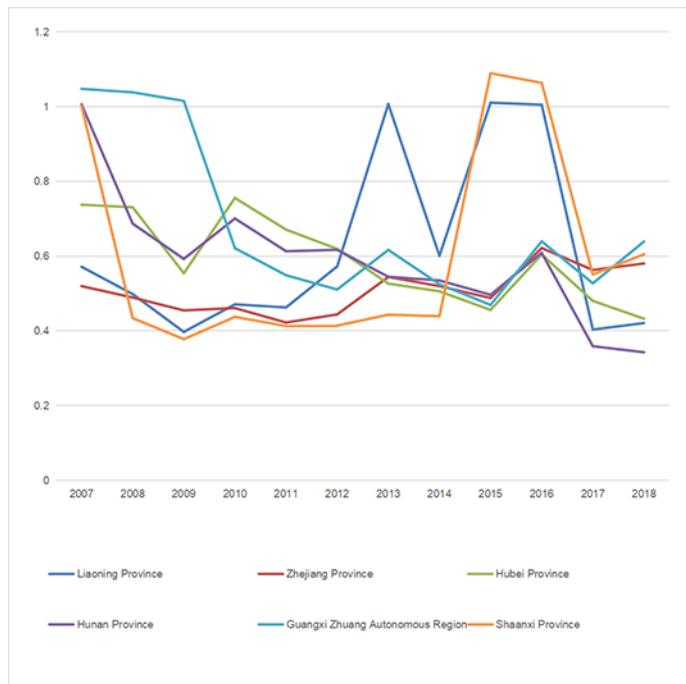

Fig 2b.tif

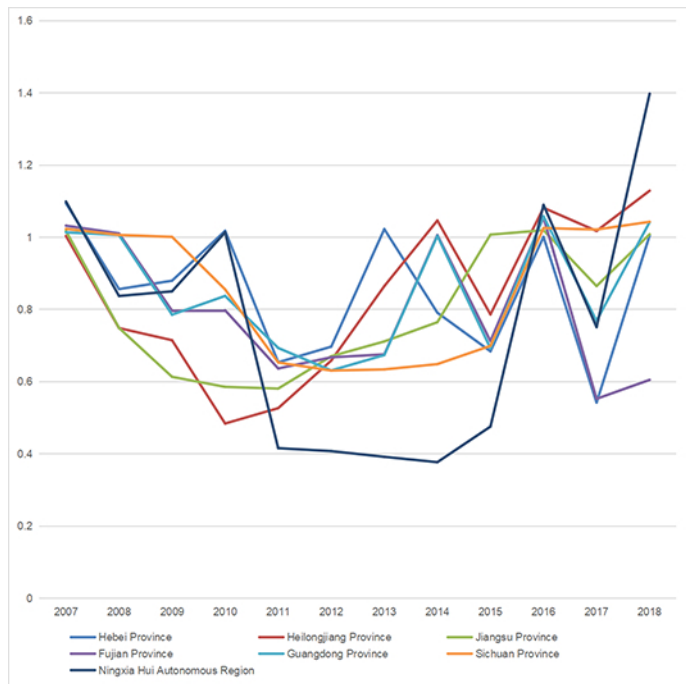

Fig 2c.tif

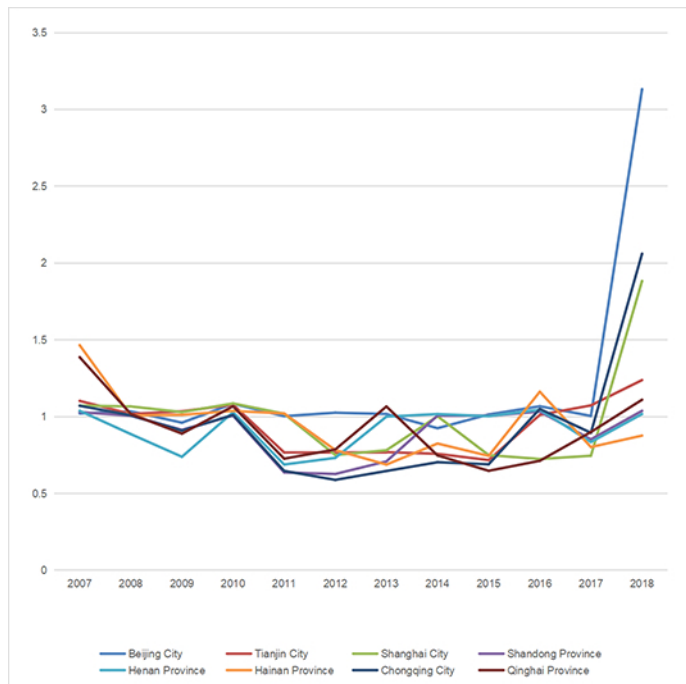

Fig 2d.tif

Supplement: S2 Raw images — (PDF) [file pone.0282051.s007.pdf]

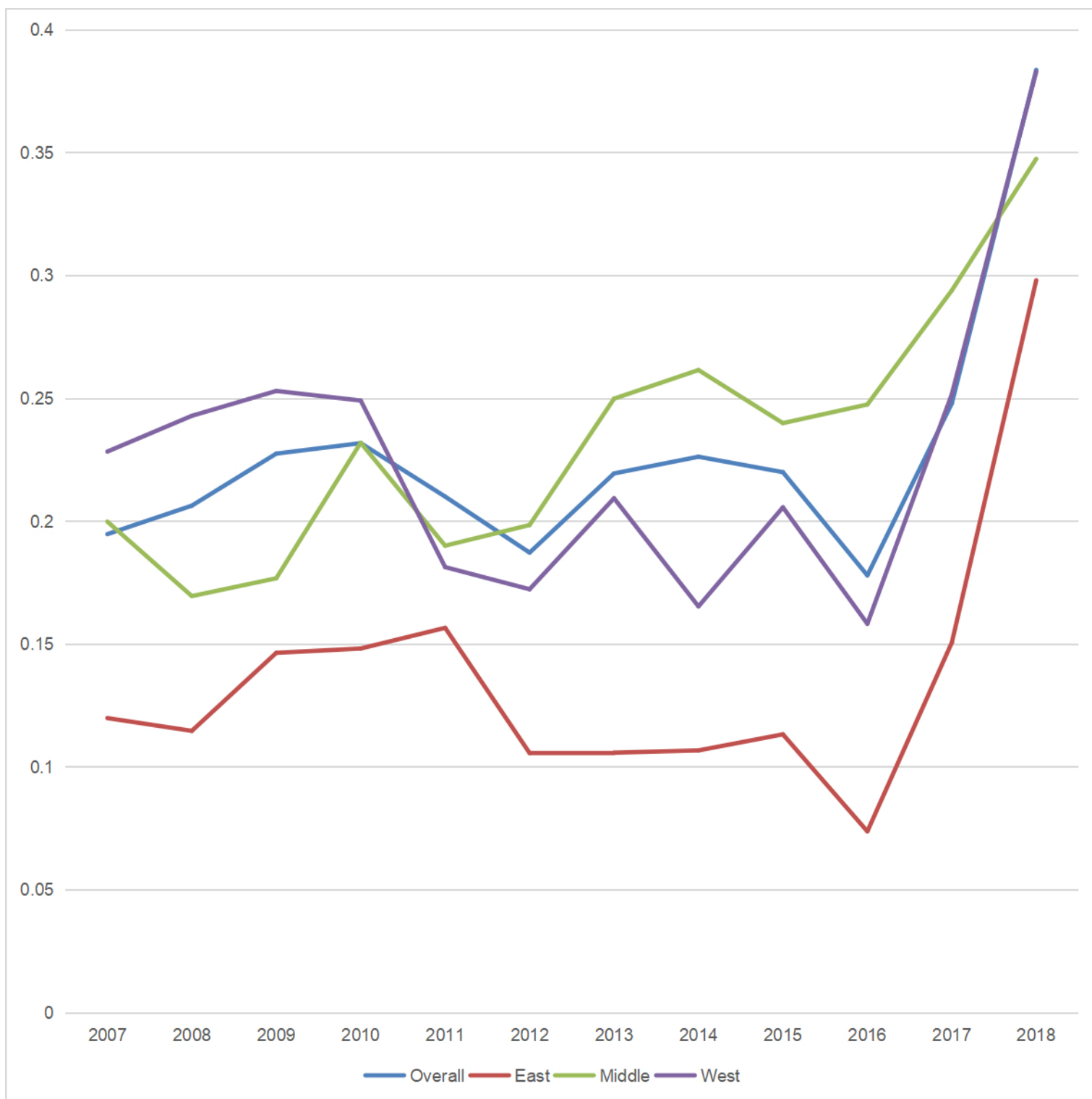

Fig 3a.tif

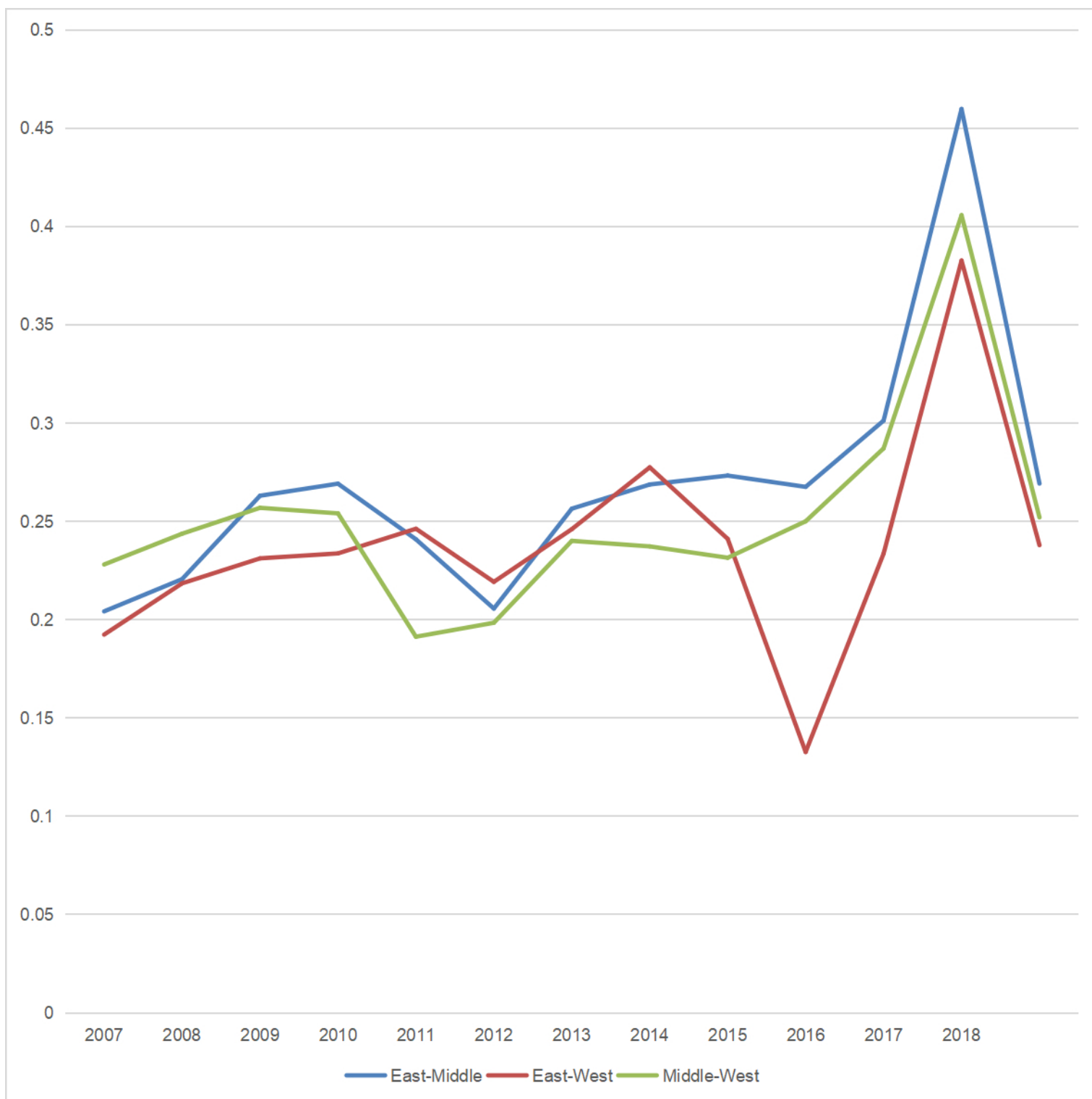

Fig 3b.tif

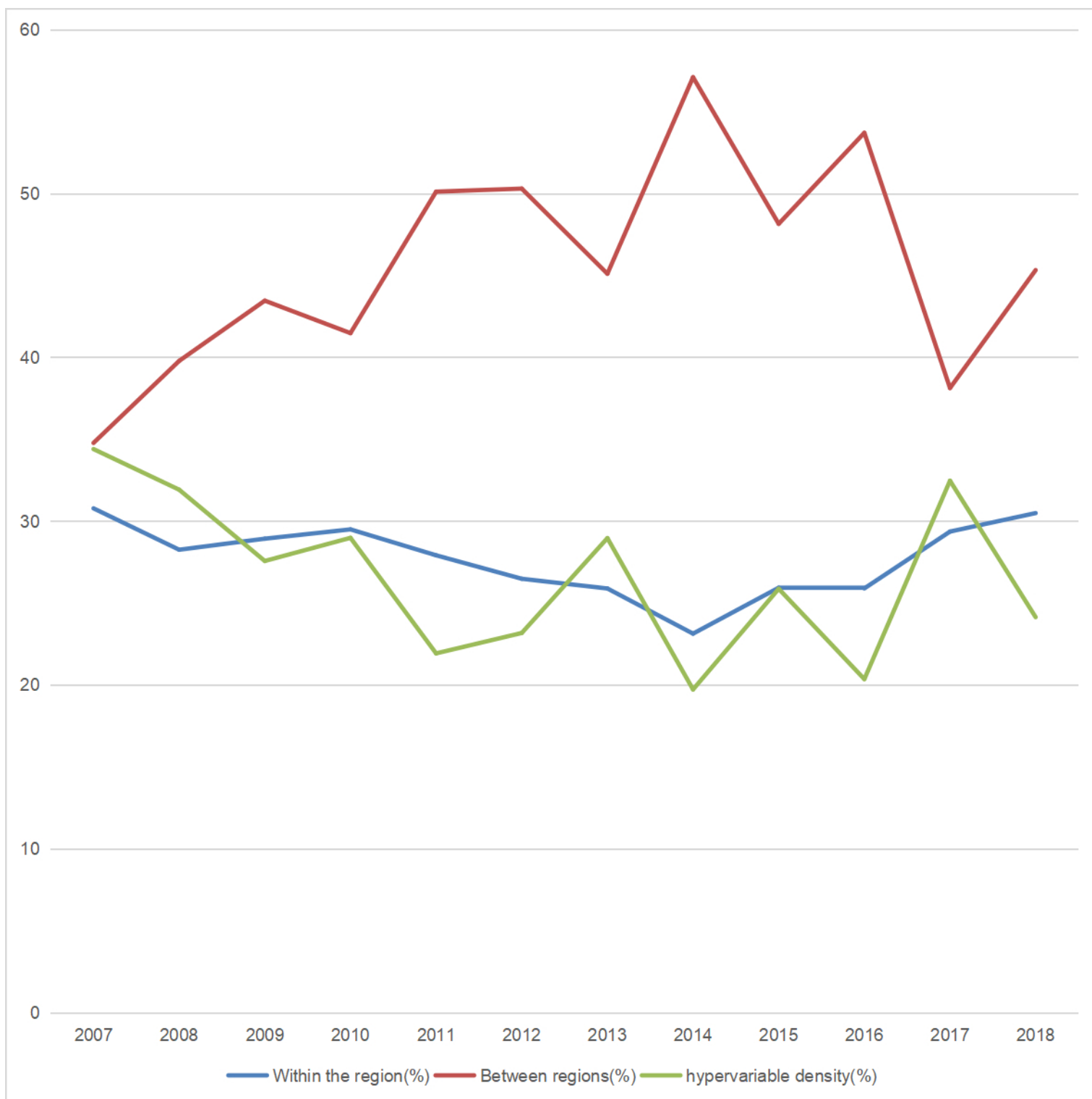

Fig 3c.tif

Supplement: S3 Raw images — (PDF) [file pone.0282051.s008.pdf]

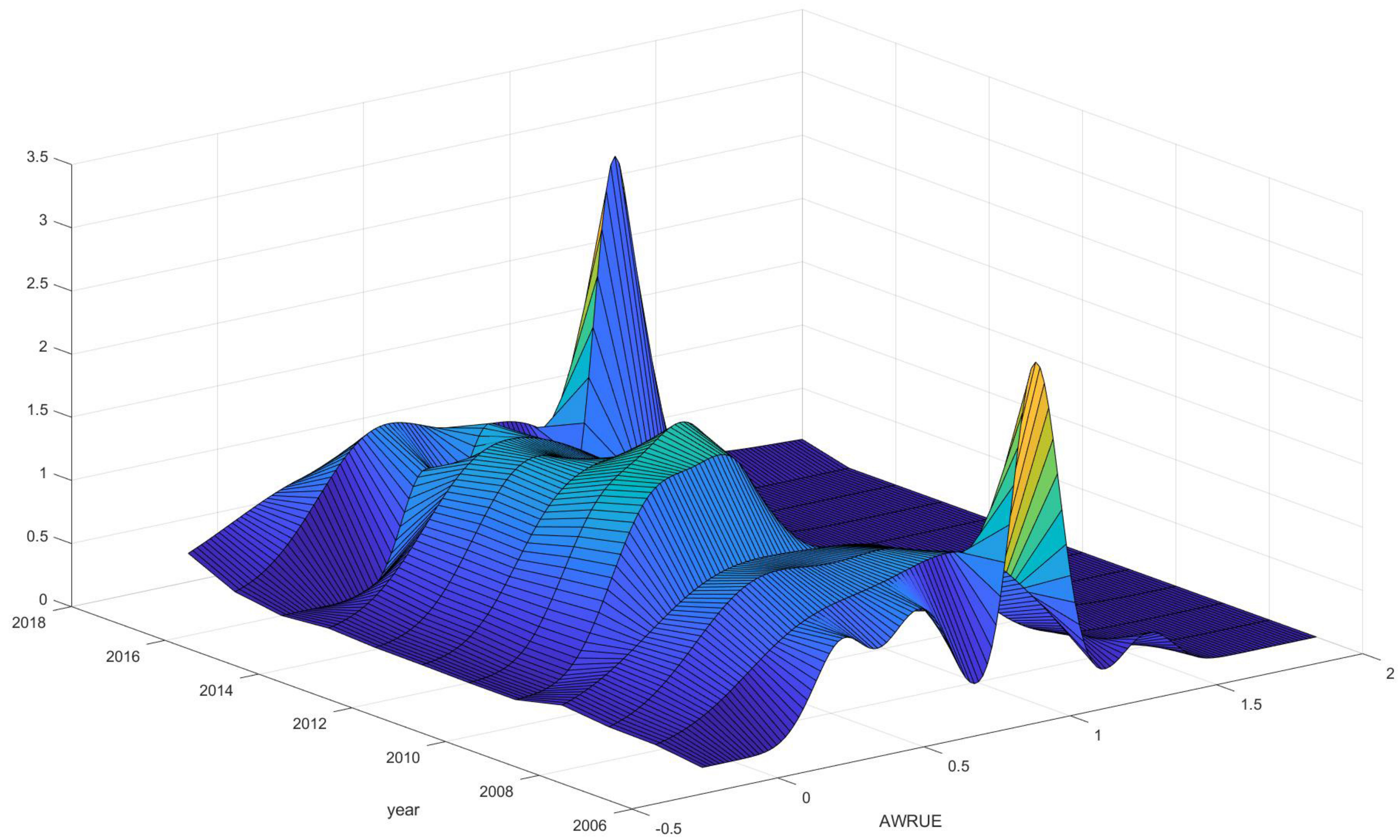

Fig 4a.tif

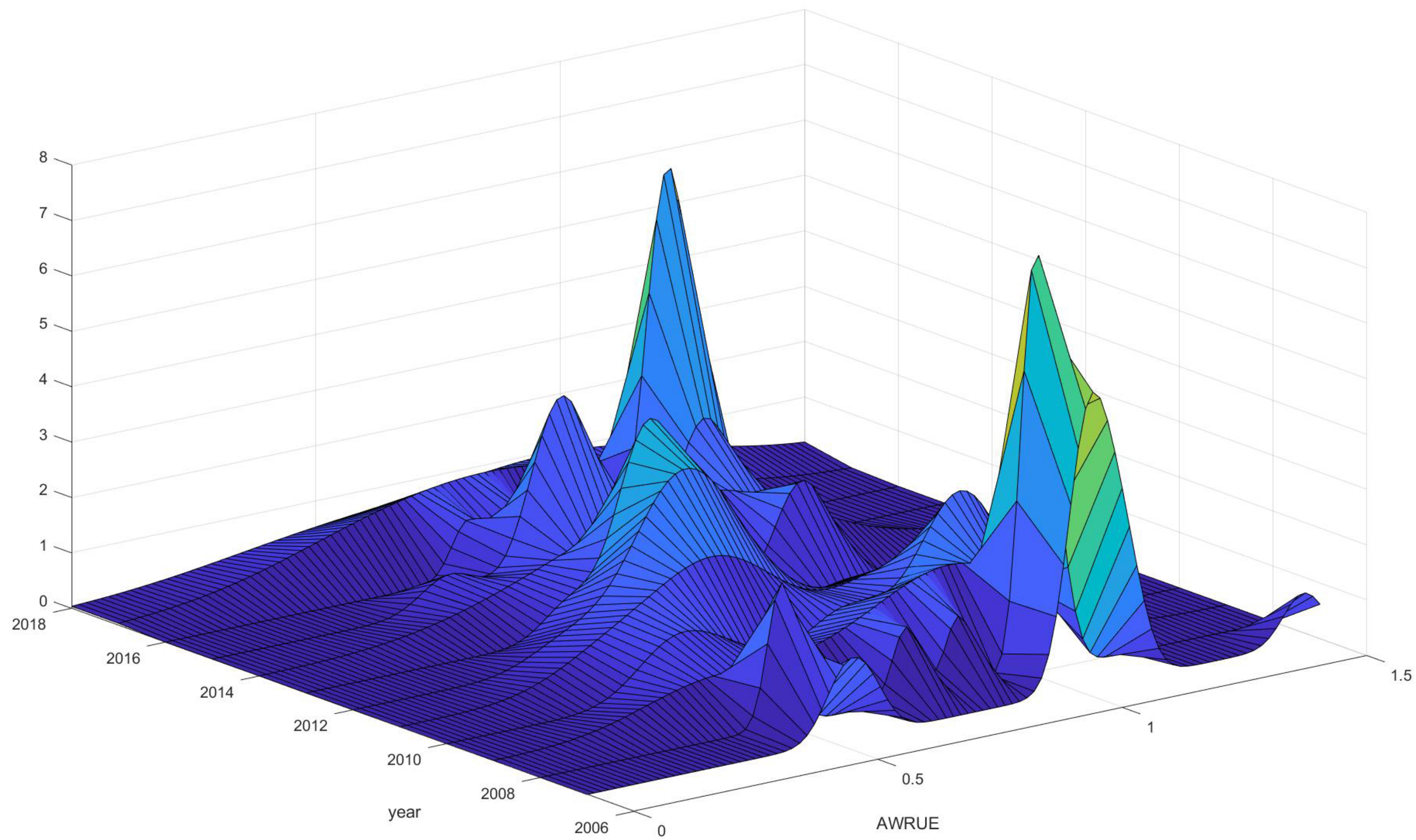

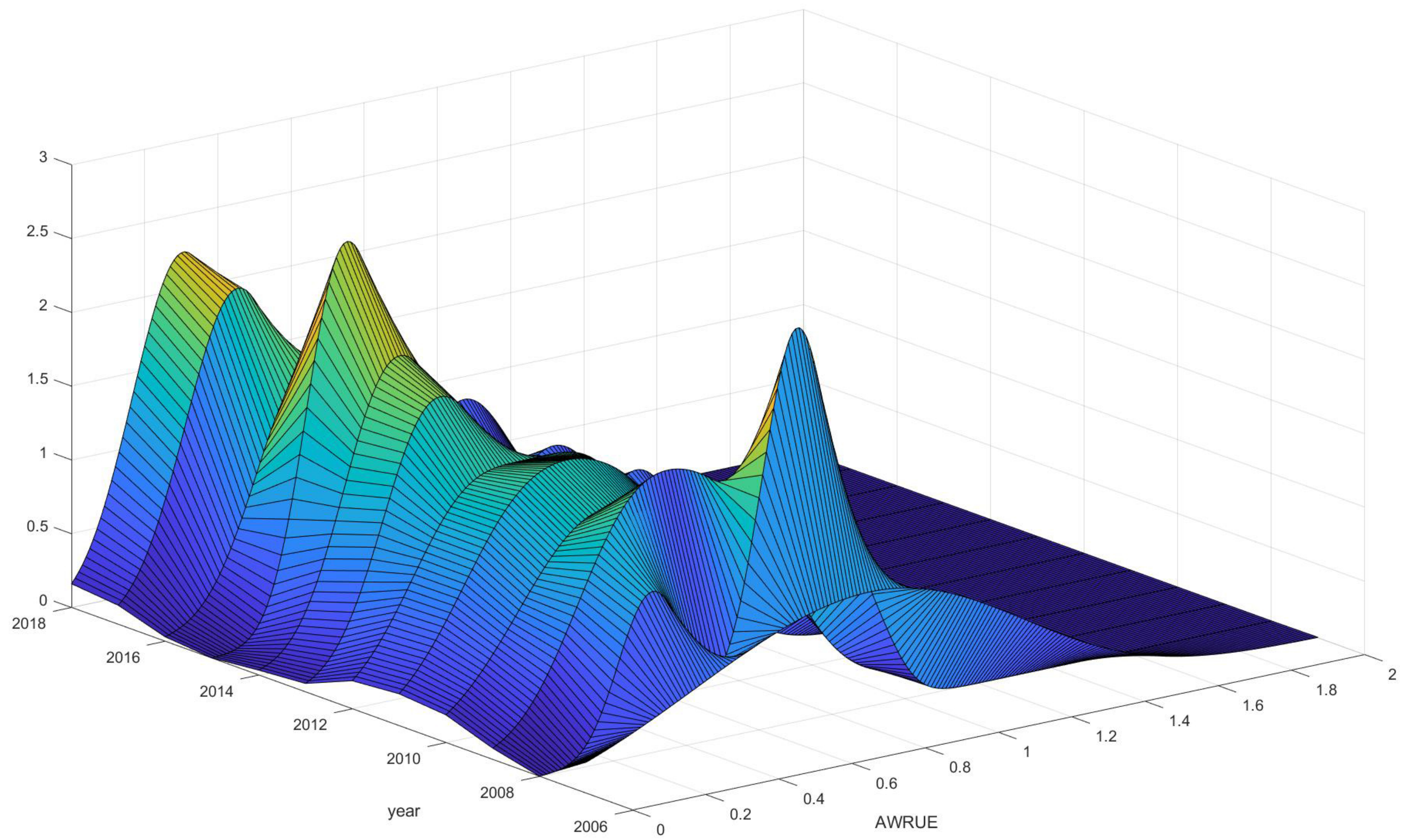

Fig 4c.tif

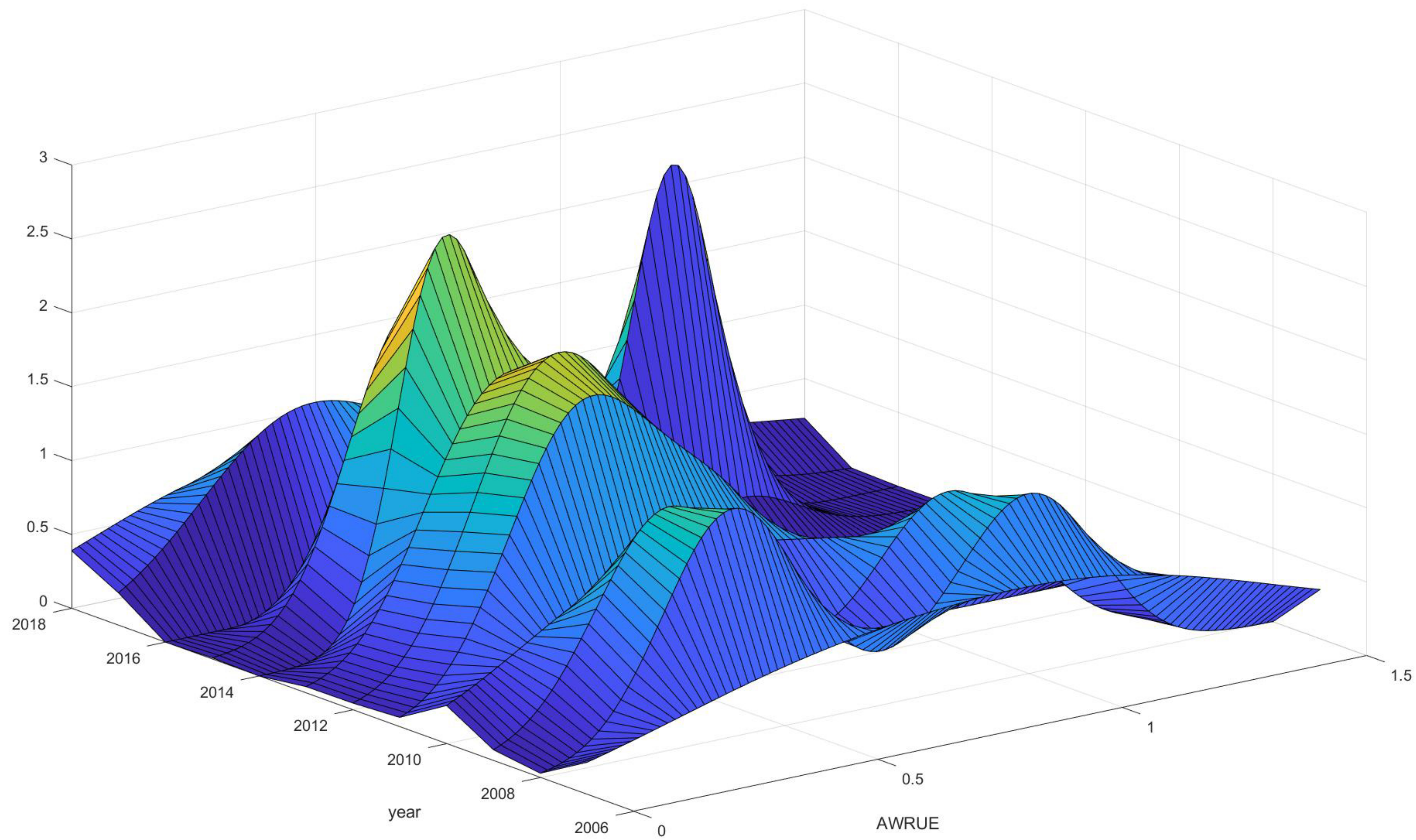

Supplement: S4 Raw images — (PDF) [file pone.0282051.s009.pdf]
