# Supplementary material for: Regional differences and dynamic evolution of agricultural water resources utilization efficiency in China
Source: PLoS One. 2023 Sep 28;18(9):e0282051. doi: 10.1371/journal.pone.0282051 (PMC10538784; doi:10.1371/journal.pone.0282051)
Supplement: Attachment — Submitted filename: Response to Reviewers.pdf [file pone.0282051.s010.pdf]

Journal:PLOS ONE
Date:NOV.13,2022
Dear Reviewers:

Thank you for your letter and the reviewers’ comments on our manuscript entitled "Regional Differences and Dynamic Evolution of Agricultural Water Resources Utilization Efficiency in China" . Those comments are very helpful for revising and improving our paper, as well as the important guiding significance to other research. We have studied the comments carefully and made corrections which we hope meet with approval. The main corrections are in the manuscript and the responds to the reviewers’ comments are as follows (the replies are highlighted in blue).
Replies to the reviewers’ comments:

Reviewer#1:

1.Line 80 - Define SBM acronym with first use.
Response：When using SBM for the first time, note the specific meaning in parentheses.It is changed to SBM (Slacks-Based Measure) in line 52.
2.Line 152 - Equation 2 or 3?
Response：It is Equation 3 in line 139.
3.Lines 154, 161-165, and throughout – Please review terms for proper use of subscripts for clarity.
Response：We have marked the subscripts correctly and make it clear.
4.Line 245 – I’m not sure what you mean by “backward” here, please clarify.
Response：“backward” is not properly used and We have replaced it with the word “lagging” .
5.Line 313 – These plots need a more thorough description in the caption along with larger fonts and clearly defined axes. I found the results in this section very interesting, but it would increase readability to have very clear plots that the reader can interpret for fully detailed results, allowing you to focus on the key results in the text.
Response：We have enlarged Figure 4 so that the axes and values can be clearly seen.
6.Lines 333-335 – Again, this is a great point of discussion, but gets a little lost in the long format presentation of results. Integrating results and discussion together is an option, but if discussion is to be held separately, this should be relocated or reiterated there.
Response：We have added a discussion section and divided it into three subsections in line392-453.

Reviewer#2:

1. At present, some literatures (such as Deng et al., 2016; Huang et al., 2021) have used SBM-DEA model to study water resource utilization efficiency in China. This paper also uses SBM-DEA model, so the author needs to clearly point out the innovation of this paper.
Deng, Guangyao, Lu Li, and Yanan Song. "Provincial water use efficiency measurement and factor analysis in China: based on SBM-DEA model." Ecological Indicators 69 (2016): 12-18.
Huang, Yujie, et al. "A study on the effects of regional differences on agricultural water resources utilization efficiency using super-efficiency SBM model." Scientific reports 11.1 (2021): 1-11.
Response：We elaborate on the innovation of the paper in line55-72.
2.It is recommended to list the existing literature as the basis for variable selection in Section 2.3 of the paper.

Response：We list the existing literature as the basis for variable selection in Section 2.3.

3.It is suggested to divide section 3 into 3.1 and 3.2, where 3.1 is the calculation result of water resource utilization efficiency (based on super efficiency SBM-DEA model) and 3.2 is the regional difference of water resource utilization efficiency (based on Dagum's Gini Coefficient).

Response：We have divided section 3(Regional Differences in Agricultural Water Resources 200 Utilization Efficiency in China) into 3.1 and 3.2,where 3.1 is the calculation result of water resource utilization efficiency (based on super efficiency SBM-DEA model) and 3.2 is the regional difference of water resource utilization efficiency (based on Dagum's Gini Coefficient).

4.Kernel Density Estimation and Markov Chain are both used to analyze the change trend of water resource utilization efficiency. It is suggested that the author discuss the relationship and difference between them.

Response：We have discussed the relationship and difference between Kernel Density Estimation and Markov Chain in line 60-72.

5.The author is suggested to add a discussion section, which is used to compare the similarities and differences between the research results of this paper and those of the existing literature.

Response：We have added a discussion section and divided it into three subsections in line 392-453.References are also attached to the discussion.

Once again, thank you very much for your constructive comments and suggestions which would help us both in English and in depth to improve the quality of the paper.Wish you a happy life!

Kind regards，

Shuya Cao
Email:caoshuya2022@163.com

Corresponding author: Shuya Cao
Email address:caoshuya2022@163.com
